# Chondroid Tenosynovial Giant Cell Tumor of the Temporomandibular Joint: A Case Report

**DOI:** 10.7759/cureus.109971

**Published:** 2026-05-31

**Authors:** Hunter D Stearns, Tanya Rath, Salvatore Lettieri, Nicholas Deep

**Affiliations:** 1 Otolaryngology, Mayo Clinic, Phoenix, USA; 2 Neuroradiology, Mayo Clinic, Phoenix, USA; 3 Plastic and Reconstructive Surgery, Mayo Clinic, Phoenix, USA

**Keywords:** chondroid tumors, temporal bone tumor, temporomandibular joint tumor, tenosynovial giant cell tumor, tmj

## Abstract

We present a rare case of a chondroid tenosynovial giant cell tumor (TGCT) involving the temporomandibular joint (TMJ) in a 46-year-old man with an asymptomatic but progressively enlarging lesion centered within the zygomatic air cell tract superior to the TMJ.

A computed tomography (CT) scan performed for sinusitis symptoms revealed an expansile lesion near the zygomatic air cell tract above the TMJ, which had grown undetected over 16 years, eroding the squamous temporal bone and extending to the middle fossa dura. Magnetic resonance imaging (MRI) showed T1- and T2-hypointense, heterogeneously enhancing nodules in the TMJ, with susceptibility artifacts on diffusion-weighted sequences indicating hemosiderin staining, suggestive of a chondroid TGCT originating from the synovial disc in the glenoid fossa. A preauricular infratemporal approach enabled complete resection of the tumor, synovial disc, and adjacent affected bone. The mandibular condyle was unaffected and preserved. Reconstruction was performed using a pedicled temporoparietal fascia flap. One-year follow-up MRI showed no recurrence, and the patient experienced successful mastication without pain.

This case illustrates the diagnosis and surgical management of chondroid TGCT of the TMJ, emphasizing its potential for slow, asymptomatic growth and its CT appearance of a primary temporal bone lesion. The characteristic MRI features assist in diagnosis, and chondroid TGCT should be considered when evaluating erosive lesions near the glenoid fossa.

## Introduction

Tenosynovial giant cell tumors (TGCTs) are rare benign tumors arising from the synovium of joints, bursae, and tendon sheaths. TGCTs can be subdivided into localized and diffuse types based on their pattern of growth and clinical course. While these tumors most commonly affect the knees and hips, temporomandibular joint (TMJ) involvement is rare, with fewer than 130 cases documented [1].

A distinct subtype of TGCT is chondroid TGCT, formerly known as pigmented villonodular synovitis (PVNS), which preferentially involves the TMJ and is even rarer, with only approximately 30 cases involving the TMJ extensively documented [2]. The rare incidence and often indolent clinical course of chondroid TGCT involving the TMJ pose diagnostic challenges and are often accompanied by diagnostic delays. Lesions may remain asymptomatic for extended periods, leading to progressive growth, osseous erosion, and intracranial extension before detection.

Radiographic imaging plays a critical role in diagnosis. Findings may be subtle or nonspecific early in the disease course. Computed tomography (CT) demonstrates erosive or lytic expansile changes, and magnetic resonance imaging (MRI) provides superior soft tissue characterization and reveals characteristic signal abnormalities related to hemosiderin deposition [3].

Patients with TMJ TGCT most commonly present with hearing loss, pain, headache, or preauricular swelling, with a median delay in diagnosis of 24 months from symptom onset [3,4]. Surgical management typically involves complete resection, and when the mandibular condyle is involved, condylectomy with reconstruction using an alloplastic TMJ prosthesis is often required. We report a unique case of chondroid TGCT of the TMJ and discuss its surgical management.

## Case presentation

A 46-year-old man was evaluated for an asymptomatic lesion on his right TMJ, which was incidentally discovered during a CT scan for sinusitis symptoms, prompting a referral to our tertiary care neurotology skull base clinic.

Compared to a study performed 16 years prior, CT demonstrated new, rounded, clustered radiolucent erosions of the squamous right temporal bone just above the mandibular fossa within the zygomatic air cell tract, extending superiorly to the middle fossa dura, inferiorly into the glenoid fossa, and anteriorly to the articular eminence (Figure 1).

**Figure 1:**
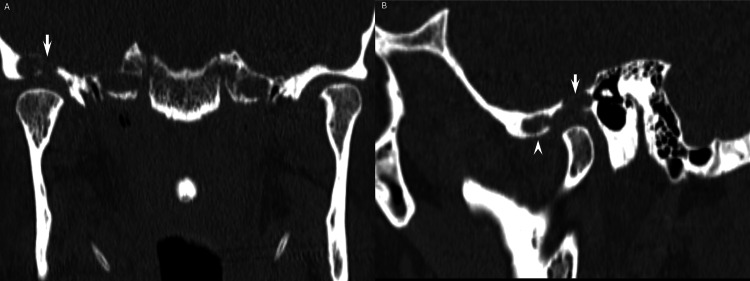
Coronal (A) and sagittal (B) maxillofacial CT images demonstrate right temporomandibular joint space widening with rounded erosive changes involving the squamous temporal bone overlying the mandibular fossa and extending to the right middle cranial fossa floor (arrow) and the right articular eminence (arrowhead)

Ipsilateral joint space widening suggested a process arising from the TMJ. MRI demonstrated T1- and T2-hypointense, heterogeneously enhancing nodules arising from the TMJ, indicating a proliferative synovial process (Figure 2).

**Figure 2:**
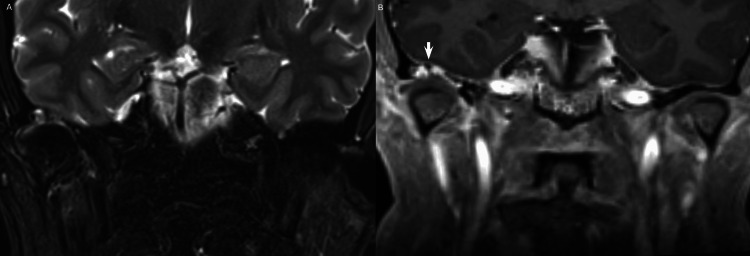
Coronal T2 fat-suppressed (A) and T1 MPRAGE contrast-enhanced (B) images demonstrate that the temporomandibular joint space widening is due to heterogeneously enhancing synovial proliferation, which erodes the middle cranial fossa floor and subtly elevates the overlying dura (arrow). There is no edema in the overlying temporal lobe. MPRAGE: Magnetization-Prepared RApid Gradient-Echo.

The associated susceptibility artifact on the diffusion-weighted sequence, characteristic of hemosiderin staining, suggested the diagnosis of chondroid TGCT of the TMJ (Figure 3).

**Figure 3:**
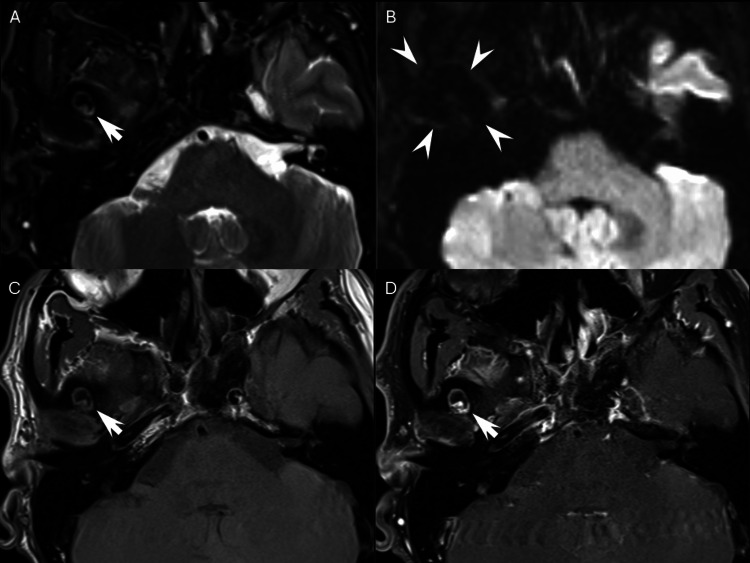
Axial T2 fat-suppressed (A), diffusion-weighted (B), T1 (C), and T1 fat-suppressed contrast-enhanced (D) images demonstrate T1- and T2-hypointense, heterogeneously enhancing synovial proliferation (arrows). Susceptibility artifact causing signal loss (arrowheads) from hemosiderin staining, a characteristic feature of tenosynovial giant cell tumor, is seen on all images but is accentuated on the diffusion-weighted sequence.

A multidisciplinary tumor board, including a neurotologist, oral-maxillofacial surgeon, plastic surgeon, and neuroradiologist, recommended surgical resection given the young age of the patient and the tumor's progressive growth nearing the middle cranial fossa.

At surgery, a preauricular infratemporal approach was used for exposure (Figure 4, Panel A). The zygomatic process of the temporal bone was visualized. The tumor was immediately seen emanating from the joint space cephalad to the articulating disc. A capsulotomy was conducted, and the condylar head appeared to be free of the tumor. The inferior aspect of the articulating disc was also free of tumor (Figure 4, Panel B). The zygomatic process of the temporal bone was removed. With the squamous temporal bone exposed, the cortex overlying the zygomatic air cell tract was drilled off, exposing the tumor.

**Figure 4:**
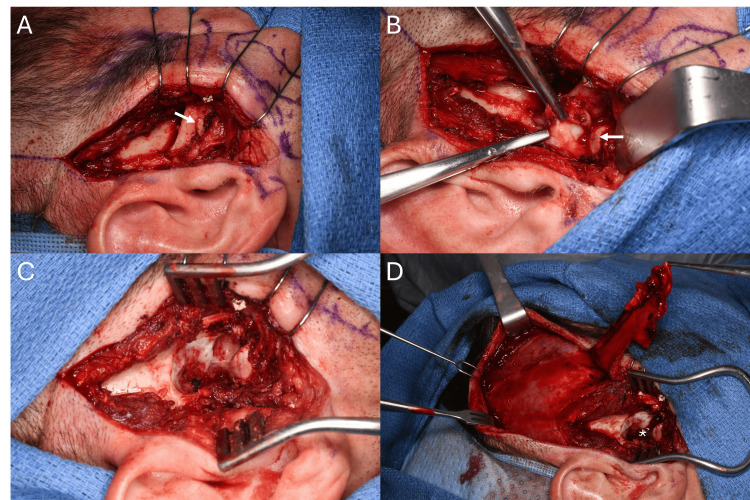
Intraoperative photos (A) After exposure through a preauricular incision, the tumor (arrow) can be seen extending from the glenoid fossa under the zygomatic arch. (B) The zygomatic arch has been removed. The articular disc is being lifted with forceps, and its inferior surface appears clear of tumor. The condyle of the mandible (arrow) can be seen. (C) The defect is shown after completion of the tumor removal, including resection of the articular disc, the soft tissue within the glenoid fossa, and involved portions of the squamous temporal bone. The condyle of the mandible (arrow) was preserved as it was not involved by the tumor. (D) A vascularized pedicled temporoparietal fascia flap has been harvested and is being held with forceps. This is subsequently rotated into the defect (asterisk) between the skull base and the condyle.

Wide exposure of the tumor was achieved​, tracing its extension from the glenoid fossa through the squamous temporal bone to the middle fossa dura. Notably, the tumor involvement of the middle fossa dura was limited to a few focal areas. Care was taken to leave the remaining bone overlying the temporal lobe intact to prevent the risk of encephalocele. The tumor remained extradural throughout the procedure and was successfully excised without compromising the integrity of the dura. The middle ear ossicles and tympanic membrane were not involved.

Attention was then directed to the tumor within the condylar fossa, which had extended anteriorly and medially, creating defects in the bone. The tumor tissue in these areas was soft and was effectively curetted away. The bone was thoroughly drilled to mitigate the risk of recurrence. The surgical resection included a complete synovectomy, removal of all tumor-involved bone in the squamous temporal area, and clearing of the glenoid fossa contents above the disc level (Figure 4, Panel C). The mandibular condyle was not involved by the tumor and was therefore preserved.

Following complete resection of the tumor, a vascularized pedicled temporoparietal fascia flap was rotated into the defect as an interpositional arthroplasty (Figure 4, Panel D). Maxillomandibular fixation with Erich arch bars and subsequent therapeutic guiding elastics was performed to achieve normal occlusion.

Final pathology confirmed a giant cell-rich tumor consistent with chondroid TGCT. Postoperatively, the patient recovered well without complications, requiring a brief return to the operating room (OR) to adjust maxillomandibular fixation and guiding elastics for optimal dental occlusion. One-year follow-up MRI showed no signs of recurrence, and the patient reported successful mastication and absence of pain.

## Discussion

TGCT, previously termed PVNS, is a rare yet locally aggressive disease predominantly affecting the synovium of joints, bursae, or tendon sheaths. This pathology primarily occurs in individuals between their second and fifth decades of life, with a median age of 47 years at diagnosis, and women account for more than half of those diagnosed with TGCT [5]. While the etiology of TGCT combines inflammatory and neoplastic components, the exact triggers of this disease remain unclear, with theories ranging from genetic predispositions to inflammatory responses to unknown stimuli.

The disease manifests in two principal forms: localized (L-TGCT) and diffuse (D-TGCT), with D-TGCT being more invasive and having a higher recurrence rate because of its extensive synovial involvement. The knees and hips are commonly affected, but TMJ involvement is exceptionally rare, with only 1.8 cases per million annually reported [6]. This form of TGCT is challenging due to its potential for severe complications, including bony erosion and intracranial extension.

Early in the disease, patients may be asymptomatic, especially when the condylar head is not involved. The tumor can grow and expand into the temporal bone without causing symptoms initially. As greater degrees of TMJ involvement occur and bone destruction progresses, symptoms include otalgia, aural fullness, trismus, or a preauricular mass [7]. Middle ear involvement can result in conductive or sensorineural hearing loss and tinnitus. Tumors that extend into the middle cranial fossa can result in headaches, seizures, nausea, and vomiting. The differential diagnosis of a preauricular mass with any combination of these symptoms is significant, and with the rare nature of TGCT, it is often overlooked and not considered in the differential. Diagnosis of TGCT is therefore complex and often delayed due to its nonspecific symptoms and rare occurrence.

CT and MRI play crucial roles in the diagnosis. CT demonstrates a lytic expansile lesion near a joint with areas of erosion that are often rounded in appearance. TGCT can demonstrate high attenuation on CT due to iron deposition within the tumor. Surrounding soft tissue swelling can be present. MRI best characterizes the extent of the lesion and often suggests the diagnosis, providing a detailed depiction of synovial proliferation with associated hemosiderin deposition indicative of the disease [8]. TGCT demonstrates intermediate to low T1 signal and a characteristic low T2 signal, accentuated on susceptibility-weighted or gradient-recalled echo imaging from hemosiderin within the tumor. Enhancement is often heterogeneous. The definitive diagnosis hinges on histopathological examination, which reveals fibrohistiocytic lesions with multinucleated giant cells and occasional chondroid metaplasia in chondroid subtypes of TGCT.

Management ideally requires complete surgical resection and consideration of reconstruction. In cases of diffuse TGCT, a total synovectomy is often necessary. A mandibular condylectomy and joint replacement may or may not be needed, depending on the involvement of the mandibular condyle. Preoperative planning should include CT and MRI to map the tumor extent and anticipate potential surgical challenges. Given the tumor's invasive growth pattern, it often creates subtle bony erosions and tracks through these defects to adjacent sites, where it may then expand.

Due to the invasive nature of TGCT, leaving these tumors untreated can lead to significant morbidity. Kanbour et al. described a patient who was symptomatic with trismus and preauricular swelling and was found to have a TGCT of the TMJ, which wrapped around the head of the mandibular condyle with bony erosion [1]. The patient required a synovectomy and condylectomy to achieve gross total resection, followed by reconstruction with a custom alloplastic TMJ prosthesis. Yang et al. described a patient with TGCT without involvement of the condylar head but extending intracranially into the middle cranial fossa with a large cystic component [6].

In our patient, the tumor did not involve the condylar head, sparing it from resection. The surgical approach involved complete resection of the synovial disc and all tumor-involved contents of the glenoid fossa and squamous temporal bone. Vascularized tissue was used for an interpositional arthroplasty to avoid the use of an alloplastic prosthesis, which could complicate postoperative MRI surveillance. This initial step may suffice for functional outcomes, but alloplastic reconstruction could be considered in a staged fashion if complications such as malocclusion or arthritic pain develop.

This case underscores the necessity of a multidisciplinary approach for effective management of TGCT, requiring meticulous, complete surgical resection and thoughtful reconstruction to optimize functional outcomes. Recurrence rates range from 10% to 50% and are more common in D-TGCT, highlighting the need for surveillance following treatment. Postoperative radiation has been demonstrated to reduce recurrence in diffuse cases [9].

## Conclusions

This case highlights the definitive diagnosis and effective surgical management of chondroid TGCT involving the TMJ. The initially asymptomatic progression and variable growth patterns of this tumor make diagnosis challenging. TGCT should be considered in the differential diagnosis of an enlarging erosive lesion near the glenoid fossa observed on imaging. MRI plays a crucial role in confirming the diagnosis, leveraging the distinct radiographic characteristics of TGCT.
